# Scientific Items

**Published:** 1885-04-20

**Authors:** 


### SCIENTIFIC ITEMS.

**The Microphotoscope.**—Mr. R. G. Mason, of Douglas, Isle of Man, is the author of the following:

The microphotoscope consists of a pair of spectacles, eyeglasses, or an eyeglass, with one or a number of minute photographs arranged in or along the rim of the spectacles, eyeglasses, or eyeglass.

The minute photographs are placed behind suitable minute magnifying glasses, and are so arranged in or along the rims of the spectacles or eyeglasses that the eyes of the wearer may see either one or all of the photographs without moving the spectacles.

The rim in or along which the minute photographs are placed may be either the rim of the spectacles themselves or a detachable rim, which may be applied to any spectacles, eyeglasses, or eyeglass.

The minute photographs may be photographs of written or printed matter, maps, charts, views, landscapes, or any object, or from which a photograph may be taken.

Some of the uses to which the microphotoscope could be applied are the following:

For a student.—The series of microphotographs in the rims of the spectacles might consist of copies of an epitomized grammar, history, or any subject the student wished to study. Thus, the subject he was studying would be constantly before his eyes for reference in his spare moments without the trouble of carrying books about with him.

The rims containing the microphotographs being detachable, he could at any time change the subjects.

A lecturer might have the heads of his lectures photographed and placed in the rims of his spectacles: a lawyer, his briefs; a clergyman, his sermons; a bicyclist, or other tourist, maps, views, and plans of the country through which he traveled; a shopkeeper, a calendar, ready-reckoner, etc.; a timber merchant or builder, cubes, measurements, and rules; travelers on the Continent, list of foreign terms, names of articles, foreign money, tables and so on; a correspondent, an abridged dictionary of technical or difficult words; a member of Parliament, facts and figures relating to the subject of his speech; a doctor, formulæ; a public entertainer, recitations, songs, *bon-mots*, etc.; a musician, whole pieces of music; a detective, criminals wanted.

The inventor says: "It will also be evident that the microphotoscope may be applied to a variety of other uses too numerous to mention."

It will be understood that the spectacle or eyeglass frames may have in them ordinary reading-glasses when worn by those who need them, and when worn by those who do not, they may have either plain glasses or no glasses at all.—*Scientific American.*

**A Great Animal.**—A greater curiosity in the animal world can not be found than the one that creates the complaint called the itch. This parasite is only a conversion of a root of hair into an animal existence. His body and legs will be found to correspond to a hair bulb and the arms on its sides. The animal is generated when a person is so meanly fed that a hair dies. A use is made of the hair by converting it into an insect of this character. The animal will live only where it is generated, just below the cuticle.

No animal on earth will exist except where it is generated, and a departure from this law can not be found.

All that renders a creature of this character smaller than the louse of the head is the smaller size of the hair that gives it being. The form of the insects is the same.

Now, will the only considerable being on earth understand that an insect is only a better development of a plant or animal cell or tissue ? All that will ever positively confirm the belief of an existence of a soul of man or any organization, is the discovery that the organization is capable of giving the atmosphere or water the object which is developed in the organization and growth of the insect, animal or human origin. Only when this creation is discovered and understood will the human soul be understood.—*Pro. of Nature.*

**Telescopes.**—We have seen Jupiter and Saturn through the great Russian telescope of 30 inches aperture and through a telescope of 8¼ inches aperture. The difference in the two views was not so much in the size as in the brighter light thrown upon the objects in the larger telescope. The great telescope is used principally to bring out objects that are invisible in smaller ones. Thus the W[illegible]sh[illegible]n[illegible]t[illegible]n telescope was renowned by its discovery of the moons of mars. Hersc[illegible]e[illegible] discovered Uranus with his reflector of 2 feet aperture. Mr. Lassell discovered two moons of Uranus with his reflector of 2 feet aperture. Lord Rosse's reflector is used chiefly for making drawings of nebulæ and lunar scenery. The reflector of the late Professor Henry Draper was used in photographing the Great Nebular of Orion, bringing out stars of the 14th magnitude. But it takes practiced eyes and the devotion of a lifetime to detect these minute and distant objects. The directors of the great observatories are absorbed in their work. They have little time or inclination to reveal celestial wonders to ordinary observers, who are untrained to see what they see or to comprehend the abstruse calculations by which they reach results.

Astronomy is making its way to the heart of the people. A widespread interest is felt in all that partains to the heavens. It is, however, popular astronomy that is demanded. The accounts of eclipses, comets, occulations, are eagerly read, and the movements of the bright planets are followed by thousands of observers all over the country.—*Scientific American.*

**Platinum Crucibles,** on being ignited, suffer a greater or less decrease in weight when they are new, but after repeated ignition such changes no longer occur.

